# A Generative Adversarial Network-Based Image Denoiser Controlling Heterogeneous Losses

**DOI:** 10.3390/s21041191

**Published:** 2021-02-08

**Authors:** Sung In Cho, Jae Hyeon Park, Suk-Ju Kang

**Affiliations:** 1 Department of Multimedia Engineering, Dongguk University, Seoul 04620, Korea; csi2267@dongguk.edu (S.I.C.); pjh0011@dongguk.edu (J.H.P.); 2 Department of Electrical Engineering, Sogang University, Seoul 121-742, Korea

**Keywords:** image denoising, convolutional neural network, generative adversarial network, image restoration, structural loss

## Abstract

We propose a novel generative adversarial network (GAN)-based image denoising method that utilizes heterogeneous losses. In order to improve the restoration quality of the structural information of the generator, the heterogeneous losses, including the structural loss in addition to the conventional mean squared error (MSE)-based loss, are used to train the generator. To maximize the improvements brought on by the heterogeneous losses, the strength of the structural loss is adaptively adjusted by the discriminator for each input patch. In addition, a depth wise separable convolution-based module that utilizes the dilated convolution and symmetric skip connection is used for the proposed GAN so as to reduce the computational complexity while providing improved denoising quality compared to the convolutional neural network (CNN) denoiser. The experiments showed that the proposed method improved visual information fidelity and feature similarity index values by up to 0.027 and 0.008, respectively, compared to the existing CNN denoiser.

## 1. Introduction

Image denoising has been studied for several decades and studies on image denoising continue to be actively conducted due to its high utilization value in various applications. Specifically, image denoising plays an important role in improving the performance of image enhancement, feature extraction, and object recognition.

The ultimate goal of image denoising is to remove image noise while preserving structural information, such as the edges and details of a given noisy image. For structural information-preserving denoising, various denoising methods have been proposed. These conventional denoising methods can be categorized as model-based optimization methods and deep learning-based methods [1].

Model-based optimization methods [2,3,4,5,6,7,8] have been extensively studied and widely used for image denoising. The most popular model-based optimization methods are anisotropic diffusion (AD) [2], total variation (TV) [3], bilateral filter (BF) [4], non-local means filter (NLM) [5], block-matching and 3D filtering (BM3D) [6], and weighted nuclear norm minimization (WNNM) [7]. In the case of AD and TV, noise elimination is performed based on the pixel-wise similarity between the current pixel and its neighboring pixels in a given noisy image. NLM, BM3D, and WNNM restore a given noisy image by using non-local similarity (NSS) which is based on the patch-wise similarity between the current patch and the other patches in a given noisy image. These NSS-based denoising methods significantly improve the quality of image denoising compared to the pixel-similarity based methods, but the computational complexity is also greatly increased.

Recently, deep learning methods [1,9,10,11,12,13,14,15,16] using clean-noisy image pairs have been widely exploited due to the rapid development of deep learning technology. In [9], the multi-layer perceptron (MLP) for image denoising was proposed. In addition to this, various deep learning methods based on the convolutional neural network (CNN) have been proposed. The most popular CNN-based methods are denoising convolutional neural networks (DnCNN) [10], and image restoration convolutional neural networks (IRCNN) [1]. These CNN-based methods greatly enhance the performance of image denoising compared to the model-based optimization methods by using the CNN-based end-to-end transformation.

Of the existing image denoising methods, BM3D, WNNM, DnCNN, and IRCNN provide excellent performances of image denoising, but are all still limited in terms of the effective preservation of structural information, such as texture and weak edges. In addition, CNN-based methods have very high computational complexities, requiring the multiplication of several tens of thousands for the convolution processes.

In this paper, we propose a new generative adversarial network (GAN)-based denoiser to improve the quality of detail preservation by using the heterogeneous losses, consisting of the structural loss and the mean squared error (MSE)-based loss. The balance of these losses is adjusted by the gradient fidelity between the original and restored images, which is estimated by the discriminator of the GAN [17] during the training process. As a result, it is possible to maintain the quality of noise suppression while restoring the structural information to be most similar to that of the original image from the viewpoint of the discriminator. In addition, we greatly reduce the computational complexity of the proposed GAN denoiser compared to that of the existing CNN denoisers. The main contributions of this work are summarized as follows:

A new GAN denoiser is proposed to improve the restoration quality of structural information by incorporating the discriminator-based gradient fidelity, and the MSE-based loss. Specifically, unlike existing methods, the proposed discriminator uses gradient values as an input to effectively estimate the structural fidelity between the original and restored images. The balancing parameter for the gradient fidelity with the MSE-based loss is adjusted depending on the estimation result by the discriminator. This means that the balancing power for heterogeneous losses can be adjusted by considering the optimal denoising direction of the input image and it leads the best reproduction of the structural information of the original image. In addition to the heterogeneous losses, we propose a new structure of GAN denoiser that can reduce the computational complexity while providing improved denoising performance by using the capsulized depth-wise separable convolution (DSC) [18] with the dilated convolution and symmetric skip connection (DSDC) compared to the existing CNN denoisers [1,10,11].

## 2. Materials and Methods

### 2.1. Overall Architecture

**Figure 1:**
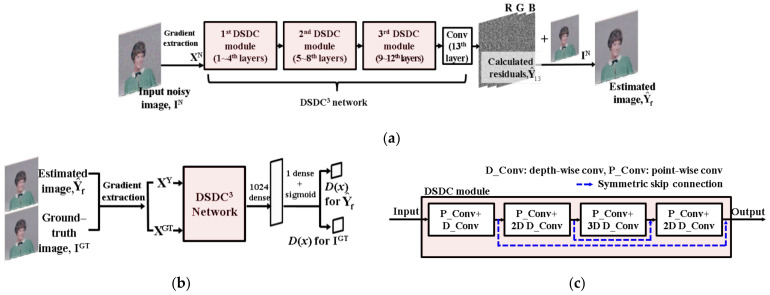
Overall architecture of the proposed GAN denoiser: (**a**) generator, (**b**) discriminator, and (**c**) element module of the proposed method (depth-wise separable convolution using dilated convolution and symmetric skip connection (DSDC)).

Figure 1 shows the overall architecture of the proposed GAN denoiser. The proposed method consists of a generator (G) and a discriminator (D), same as the conventional GAN [17]. In our work, we utilize this training approach with the heterogeneous losses, which will be described in Section 2.3.

As shown in Figure 1, in the proposed GAN denoiser, the G and the D have 13 convolution layers. To improve the denoising quality of the proposed network, we use multiple symmetric skip connections (DSDCs) that are the element modules of the proposed network and contain the dilated convolution–based depth-wise separable convolution (DSC) [18] and symmetric skip connection between two dilated convolutions having the same dilation size as shown in Figure 1c. The G uses the CNN structure based on an end-to-end transformation. In the case of the D, the two fully connected layers are added to the last convolution layer so that the scalar probability value indicating whether the input image is a noise-free image can be derived. For the input of the G and the D, gradients of a given input are used, as shown in [19]. The gradients are extracted from eight neighborhoods of a current pixel, and there are three kinds of color channels. Therefore, the total 24 feature channels are used as inputs for the G and the D. In the case of the G, this gradient input can increase its denoising performance [19]. In the case of the D, this can help the D estimate the fidelity of structural information between the restored and the noise-free images.

### 2.2. Architecture of the Proposed Network

#### 2.2.1. Generator

The general CNN architecture that uses the end-to-end transformation is used for the G in the same way as the existing CNN denoisers [1,10]. Batch normalization (BN) [20] and the ReLU [21] are selectively applied to each layer as in shown Table 1. In this table, Conv in the 13th layer represents the general convolution operation. P_conv and D_conv represent a point-wise convolution and a depth-wise convolution, respectively.

When the D_conv is applied, the dilated convolution [22] is used to increase the receptive field of convolution operation. Since each feature channel to which the D_conv is applied already contains the combined result of the previous F_D1_ feature maps, the dilated convolution is a very effective approach for enhancing the denoising performance. Compared to the existing networks that increase the size of the dilation in half of the entire layers and then decrease it in the rest layers [1,10,19], the proposed method uses the DSDC module to create multiple cycles that repeat the expansion and contraction of the dilation size to prevent the artifacts that may occur as the size of the dilation becomes too large. Also, the symmetric skip connection can increase the efficiency of information transfer between dilated convolutions. In our method, the number of layers, F_D1_, and F_D2_ were set to 13, 96, and 96, respectively, by considering the total number of weights for convolution and the quality of image denoising. Specifically, we determined the numbers of layers and F_D1_, F_D2_ values so that only the smaller number of parameters than existing CNNs can be used while providing a comparable to or better denoising quality than the existing CNNs [1,10,19]. Even if the number of convolution layers, F_D1_, and F_D2,_ are increased, the computational complexity of the proposed method is much smaller than that of the conventional CNN denoisers [1,10] which use the general convolution process because the decrease in computational complexity by the DSC overwhelms the increase in the computational complexity by the increase in the number of layer, F_D1_, and F_D2_. In particular, the selected values of F_D1_ and F_D2_ are determined by analyzing the variations in the denoising performance against the increases in F_D1_ and F_D2_ values. The generation of each convolution layer by the DSC and Conv can be formulated as follows:

(1) $${\hat{Y}}_{L}=\left\{\begin{array}{cc} \max\left(W_{L}^{D}*\left(W_{L}^{P}*X^{N}\right)+bias_{0},0\right) & if\;L=1\\ \max\left(BN\left(W_{L}^{D}*\left(W_{L}^{P}*{\hat{Y}}_{L-1}\right)\right),0\right) & \begin{array}{l} else\;if\;\\ 2\leq L\leq 12 \end{array}\\ W_{L}*{\hat{Y}}_{12}+bias_{13} & otherwise \end{array}\right.,$$

where L is an index for the convolution layer. ${\hat{Y}}_{L}$, and **bias_L_** are the Lth resultant convolution layer and the bias for the Lth convolution, respectively. **X^N^** is the input data of the proposed network. In our work, **X^N^** is the 24 kinds of gradients of the input noisy image, as shown in [19]. $W_{L}^{P}$ , $W_{L}^{D}$, and **W_L_** are the weight sets for Lth P_conv, D_conv, and Conv, respectively, and *BN* is the batch normalization operator. In addition to this convolution operation, symmetric skip connection is used as in Figure 1c. For the training of the proposed network, residual learning [23] that trains the network to convert a given input data to the residual between a training input data and its ground-truth data, which denotes image noise is used. Hence, the final restored image by the *G* can be calculated by:

(2) $${\hat{Y}}_{f}=I^{N}+{\hat{Y}}_{13},$$

where **I^N^** and ${\hat{Y}}_{f}$ are the noisy and final restored images, respectively. ${\hat{Y}}_{13}$ is the final result of the *G* and denotes the negative value of image noise.

#### 2.2.2. Discriminator

In the general GAN [17], the following adversarial min-max problem is used for training:

(3) $$\begin{array}{l} \underset{G}{\min}\;\underset{D}{\max}\;E_{I^{GT}\sim P_{train}\left(I^{GT}\right)}\left[\log D\left(I^{GT}\right)\right]\\ \;\;\;+E_{I^{N}\sim P_{G}\left(I^{N}\right)}\left[\log\left(1-D\left(G\left(I^{N}\right)\right)\right)\right], \end{array}$$

where **I^GT^** and **I^N^** are a ground-truth image and an input noisy image, respectively. *P_train_* and *P_G_* are the data distributions of the ground-truth image and resultant image by the *G*. *D* (·) denotes the output of the *D*, which indicates the probability that the current input is the ground-truth. The *G*(**I^N^**) denotes the output of the *G* for a given noisy image, thus, it is the restored image from a noisy image by the *G.* Therefore, the *D* is trained so that *D*(**I^GT^**) is close to 1 and so that *D*(*G*(**I^N^**)) is close to 0.

We utilize this training process of the general GAN for the training of the proposed method. The general CNN denoiser [1,10] is trained using MSE between the ground-truth and restored images. In this case, some small structural information, such as weak edges or texture, can be lost because the training is performed only in the direction of reducing MSE of the entire image. We alleviate this problem by incorporating MSE and the gradient-based structural loss that can be adjusted by the result of the *D*. In the proposed method, the *D* uses the gradients of a given ground-truth image (**X^GT^**) and the gradients of the restored images (**X^Y^**) as an input as shown in Figure 1b so that it can estimate the restoration quality of gradient information of the *G*. (**I^GT^** and *G*(**I^N^**) in Equation (3) is changed to **X^GT^** and **X^Y^**, respectively.) For example, a high *D*(**X^GT^**) and a low *D*(**X^Y^**) indicate that the performance of gradient information restoration of the *G* is lower than the classification accuracy of the *D*. For this case, the strength of structural loss is increased for the training of the *G*, while in the opposite case, MSE-based loss is increased for the training of the *G*. Through this training strategy, the proposed GAN reproduces the structural information most similar to that of the ground-truth image while maintaining the quality of noise suppression in smooth regions. This loss function and training process used for the proposed method will be described in detail in Section 2.3.

Table 2 shows the structure of the proposed *D*. The *D* is composed of 13 convolution layers as in *G*, and BN and ReLU are applied between the two consecutive convolution layers. After the 13th convolution layer, two dense layers are connected. Finally, the sigmoid activation function is applied to extract the scalar probability value that the input image is the original noise-free image. Because the *G* is intended to deduce the original pixel value from a noisy input patch, whereas the *D* is intended to determine the probability that the input patch is the original patch, we consider that the problem difficulty of the *G* is higher than that of the *D.* Hence, we set the size of the future channel of the *D* (F_D1_ and F_D2_) to 1/3 that of the *G,* so that we can balance the performances between the *G* and the *D*.

### 2.3. GAN-Based Heterogeneous Losses Function

The GAN-based denoiser is described in Section 2.2.2. In the general GAN, training for *G* and *D* is performed using the results of *G* and *D* as described in Equation (3). For the proposed *D*, the training is performed in the same way as the training of the general GAN by maximizing loss described in Equation (3). For the proposed *G*, the training using Equation (3) can also be applied. However, this training approach is not suitable for the *G* (CNN denoiser) that transforms a given noisy image to a denoised image in an end-to-end manner. This is because the purpose of the GAN is to understand or learn an intended context of a given image and reproduce the intended context, not to accurately restore each pixel value. Hence, we propose a new heterogeneous losses function, which consists of MSE-based loss (*Loss_residual_*), GAN loss (*Loss_GAN_*), and structural loss (*Loss_struct_*). *Loss_struct_* is calculated from the fidelity of structural information between the original image and restored image by the *G*, which is estimated by the *D*. These are used as an auxiliary loss to *Loss_residual_* in order to improve the preservation quality of structural information while also increasing the overall performance of noise suppression during the training process in a stable manner as follows:

(4) $$L=\sum_{i}^{N_{T}}\left(Loss_{residual}\left(i\right)+Loss_{GAN}+\alpha \left(i\right)\cdot Loss_{struct}\left(i\right)\right),$$

where *i* and *N_T_* denote the index for training patch pairs and the total number of training patch pairs, respectively. *L* is the final loss value for the training. *Loss_residual_* denotes the residual loss, that is, MSE between the resultant image by the *G* and the ground-truth data. In our work that utilizes the residual learning, the target data of the training is the residual (difference) of an input noisy image and a noise-free image, which represents the negative value of image noise. *Loss_GAN_* is the general GAN loss denoted in Equation (3). *Loss_struct_* denotes the structural loss, that is, the dissimilarity between the gradients of a noise-free image and a resultant image by the *G*. *α* is the balancing factor for *Loss_struct_*, which can be controlled by the results of the proposed GAN. In our method, *α* indicates the inverse fidelity of structural information between the original image and the produced image by the *G*. The *α* value was determined as the value obtained by dividing the output of the *D* for the gradient values of **I^GT^** and the output *D* for the gradient values of resultant image by *G*. More specifically, the meaning of the value *α* can be said to be an index that evaluates how well the generator has preserved structural information after denoising from the point of view of a discriminator. Therefore, as the difficulty of restoring structural information increases, the *α* value increases, and the *Loss_struct_* is more reflected in the update of the convolution coefficient than the *Loss_residual_*. This value will be explained in detail in a later paragraph.

(5) $$\begin{array}{l} Loss_{residual}\left(i\right)={\left\|{\hat{Y}}_{13}\left(i\right)-R\left(I^{N}\left(i\right)\right)\right\|}^{2},\\ R\left(I^{N}\left(i\right)\right)=I^{GT}\left(i\right)-I^{N}\left(i\right), \end{array}$$

**Figure 2:**
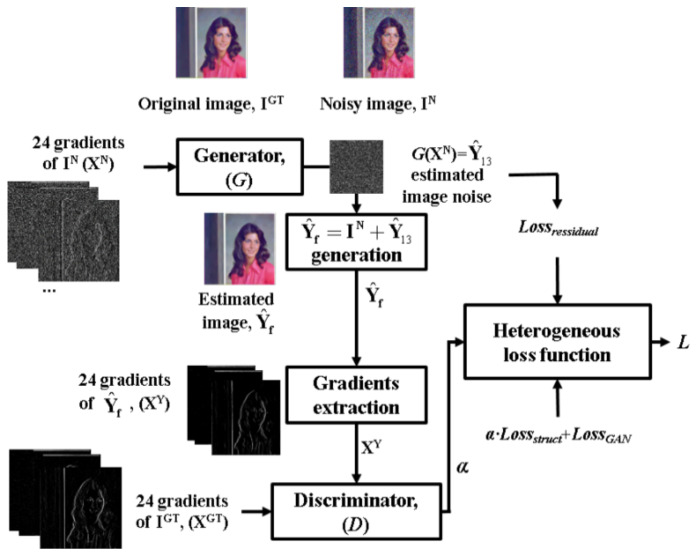
The proposed GAN-based training model using the gradient fidelity-based heterogeneous loss function.

**Figure 3:**
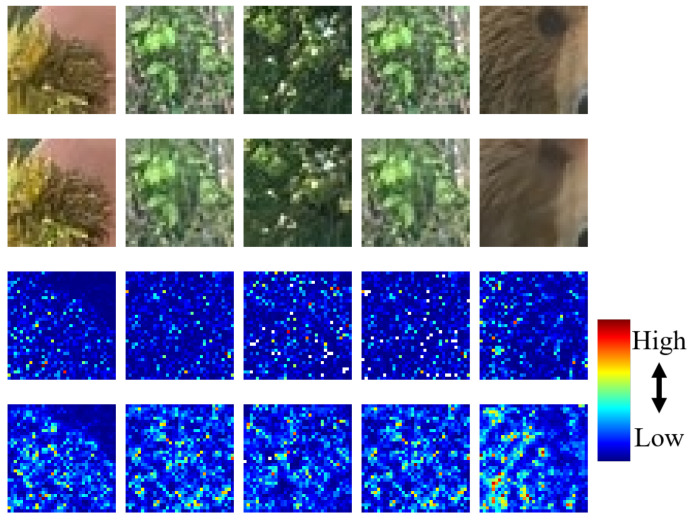
Examples of *Loss_residual_* and *Loss_struct_*: 1st row: original patches, 2nd row: resultant patches by the *G*, 3rd row: normalized *Loss_residual_*s, 4th row: normalized *Loss_struct_*s.

As shown in this equation, the *Loss_residual_* is calculated by using MSE between results by the *G* and the residual (**I^GT^** − **I^N^**) image. The *Loss_residual_* plays a key role in improving the overall performance of noise suppression in a stable manner during the training process. However, as mentioned before, some weak structural information, such as texture or weak edges, can be lost because the training of the *G* is performed in order to improve the overall pixel-wise similarity between the **I^GT^** and the restored image by the *G*. In order to alleviate this, *Loss_struct_* and *Loss_GAN_* are added to *Loss_residual_*. *Loss_GAN_* is defined as follows:

(6) $$Loss_{GAN}\left(i\right)=\left(\log D\left(X^{GT}\left(i\right)\right)+\left(1-\log D\left(X^{Y}\left(i\right)\right)\right)\right),$$

where **X^GT^** and **X^Y^** are the gradient values of **I^GT^** and ${\hat{Y}}_{f}$, as shown in Figure 2, respectively. In addition to *Loss_GAN_*, *Loss_struct_* is defined as follows:

(7) $$Loss_{struct}\left(i\right)=\left(\sum_{Dir=1}^{N_{D}}\frac{{\left\|X^{Y}\left(i,Dir\right)-X^{GT}\left(i,Dir\right)\right\|}^{2}}{\left|X^{Y}\left(i,Dir\right)\right|\cdot \left|X^{GT}\left(i,Dir\right)\right|+\lambda }\right),$$

where *Dir* indicates the direction of gradient values. *λ* is the offset value and was empirically set to 32. *N_D_* was set to 24 because gradients on eight neighborhoods of the current pixel in three color channels were used. Considering that the gradient of a given image is the most basic and important information used to derive structural information, *Loss_struct_* can effectively reflect the loss of structural information. Figure 3 shows example results of the normalized *Loss_residual_* and *Loss_struct_* of training patches. As shown in this figure, compared with the energy distribution of *Loss_residual_*, the energy distribution of *Loss_struct_* is concentrated in the texture or edge areas, having a relatively high amount of structural information in a training patch.

**Figure 4:**
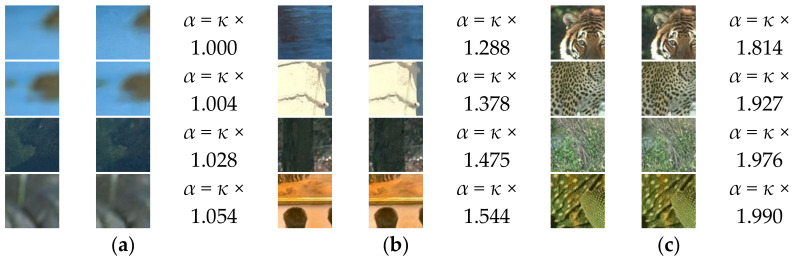
Examples of original (left) patches and resultant patches (right) by *G* with *α* values. (noise level: *σ*_n_ = 25) (**a**) *α* < = κ × 1.100 (**b**) κ × 1.100 ≤ *α* < κ × 1.600 (**c**) κ × 1.600 ≤ *α*.

Therefore, if the balance between *Loss_residual_* and *Loss_struct_* can be adjusted depending on the characteristics of the patch, the qualities of noise suppression and the preservation of structural information can be maximized. Hence, we tried to set the ideal training direction using *α* as the balancing value. To estimate the characteristics of the patch, we utilized the result of *D* as shown in Equation (4). As mentioned in Section 2.2.2, the *D* provides two probabilities (*D*(**X^GT^**), *D*(**X^Y^**)) ranging from 0 to 1. These values represent the fidelity of structural information between **I^GT^** and ${\hat{Y}}_{f}$**,** that are the original patch and restored patch by the *G.* By using these probabilities, *α* can be defined as follows:

(8) $$\alpha \left(i\right)=\kappa \times \frac{D\left(X^{GT}\left(i\right)\right)+1}{D\left(X^{Y}\left(i\right)\right)+1},$$

where *κ* is the scaling factor and was set to 3 through the extensive experiments. In our method, 1 in numerator and denominator is the offset value. If the value of *D*(**X^GT^**(*i*)) is large and the value of *D*(**X^Y^**(*i*)) is small, this indicates that it is easy to distinguish the **X^GT^**(*i*) from the **X^Y^**(*i*) from the viewpoint of the *D*. In other words, the restoration result of the gradients information by the *G* for the corresponding *i*th input training pair is not accurate, indicating that the *G* provides a restored image that has a low fidelity of structural information with respect to **X^GT^**. In this case, *α* is increased (to be closed to *κ ×* ((1 + 1)/(0 + 1)) = *κ ×* 2) so as to increase the strength of *Loss_struct_*. As a result, the training is concentrated on improving the fidelity of the structural information. In the opposite case, the accuracy of gradient restoration of the *G* is high, which leads to a decrease in *α* (to be closed to *κ ×* ((0.5 + 1)/(0.5 + 1)) = *κ ×* 1). In this case, the training is focused on smoothing-based noise suppression. Consequently, the strength of *Loss_struct_* can be continuously updated depending on the fidelity between the gradient of the **I^GT^** and ${\hat{Y}}_{f}$, and this fidelity can be estimated by the *D*. The patch including a texture region with high energy is generally more difficult to restore than the patch including a smooth region or a region having clear boundaries, thus, it is likely to have a low fidelity of structural information (as it is easy to be smoothed). These characteristics are well reflected by *α* value. Figure 4 shows examples of **I^GT^** and ${\hat{Y}}_{f}$ paired with their *α* values. As shown in this figure, the patches with relatively low energy (Figure 4a), including unclear boundaries, have low *α* values, thus the loss function with a strong *Loss_residual_* value is used for training. For this case, the strong noise suppression is performed. For the patches with clear boundaries (Figure 4b), a moderate strength of *Loss_struct_* is used for training. Finally, for the patches with texture areas (Figure 4c), a high *α* value is applied and a strong *Loss_struct_* is used for training. Hence, the texture region that is easy to be smoothed by a denoiser can be effectively preserved. The utilization effectiveness of the *α* for improving the preservation quality of structural information will be analyzed in the experimental results.

## 3. Simulation Results

Simulations for testing were performed with widely-used color testing sets, which are Kodak, misc1 (CIPR_M), and Cannon datasets (CIPR_C) from the CIPR image databases (CIPR) [24]. In addition, images captured from the IEC62087 (IEC) [25], football sequences [26], and CBSD68 dataset that is the color version of the grayscale BSD68 dataset were also used as test image sets. For the image noise model, additive white Gaussian noise (AWGN) with typical values of 15, 25, and 35 *σ_n_*s was used [1,10,19,27].

The color versions of NLM (NLM_C_) [5], block-matching, and 3D filtering (BM3D_C_) [6], which are popular image denoising methods, were used as benchmark methods. The weighted nuclear norm minimization (WNNM) [7] and Multi-channel WNNM (MCWNNM) [8] which are the recent state-of-the-art image denoising methods were also used as a benchmark method. All of these methods were simulated using publicly available MATLAB code. Other benchmark methods were the color versions of MLP [9], DnCNN_C_ [10], IRCNN_C_ [1], and MemNet_C_ [11] which are recent CNN denoisers. For the generation of denoised results of MLP, DnCNN_C,_ and IRCNN_C_, we used the already trained parameters provided by publicly available MATLAB code. For the case of MemNet_C_, we trained the model by using same environments with the proposed method. The training environments will be described below.

An Adam solver [28] was used for the training of parameters in the proposed CNN denoiser. The initial step size for each iteration of training was set to 3 × 10^−2^, and the step size was decreased to 9/10 for every 2000 iterations. The training was terminated when the loss function defined in Equation (4) no longer decreased. For the training images, we used a total of 4000 images, of which 500 were selected from the Berkeley Segmentation Dataset [29], 3000 were selected from the ImageNet database (3000 of the front images out of a total of 5500 images in ILSVRC2017 Object detection test dataset) [30], and 500 were selected from the Waterloo Exploration Database [31] (500 of the front images out of a total of 4744 images). The size of the training patch was set to 70 × 70 pixels considering the receptive field of our method, and training patches were randomly cropped from the four corners and centers of the training images. The mini-batch size for each iteration was set to 20. The proposed method was implemented using the tensor flow [32].

The performances of the proposed method with the five benchmark methods were evaluated in two ways: First, the qualities of image denoising were compared using the PSNR, structural similarity index (SSIM) [33], visual information fidelity (VIF) [34], and feature similarity index (FSIM) [35] values. Although PSNR is the most widely used objective evaluation method for image quality, it is limited in evaluating the loss of small structural information or perceptual image quality, because it is calculated by considering only the squared difference between the original pixel value and the resulting pixel value. In order to alleviate this, SSIM, which can consider the similarity of structural information between the resultant and ground-truth images, is proposed. However, SSIM is also based on MSE [36], so the difference of pixel values can dominate its resulting value rather than the fidelity of structural information for some images. Therefore, we added VIF and FSIM, which are widely used for various image processing applications [37,38,39,40,41,42,43,44] to the PSNR and SSIM as the image quality evaluation metric to accurately evaluate the quality of structural information preservation. VIF, which is based on image information fidelity measures the similarity between images by the amount of information that can be extracted by the brain from a given image. The value of VIF is equal to 1 when the resultant image is a copy of the ground-truth image. FSIM provides the feature similarity index by measuring the similarity of low-level features between resultant and ground-truth images. By using VIF and FSIM, we could more accurately evaluate the improvements obtained through the usage of GAN-based heterogeneous losses.

### 3.1. Comparisons of Denoising Quality

Table 3 shows the PSNR, SSIM, VIF, and FSIM values for the five benchmark methods and the two kinds of proposed methods, which are Pro_w/o_*D* (DSDC^3^) and Pro_wtih_D. The Pro_w/o_D (DSDC^3^) is the proposed *G* which uses the three DSDCs as shown in Figure 1a and does not use the *D* and *Loss_struct_* during training, and the Pro_wtih_D is the proposed *G* which uses the *D* with *Loss_struct_* during training. As shown in this table, except the MemNet_C_ which requires the tremendous computational complexity, the Pro_w/o_D provided the best PSNR and SSIM values for most noise levels and image sets while using a much smaller number of convolution weights than the DnCNN_C_ and IRCNN_C_. (The comparison of computational complexity will be analyzed in detail in Section 3.2). This demonstrated that the proposed DSDC^3^ network, which has a cascade structure of the three DSDCs, is a very effective convolution approach to image denoising. The MemNet_C_ provided slightly higher denoising quality than the proposed DSDC^3^ network, but it has a much higher computational complexity. For the fair comparison, we compared the proposed method with the MemNet_C_ by increasing the number of DSDC. Table 4 shows the PSNR and SSIM values of the proposed DSDC^5^ network (Pro_w/o_D_DSDC^5^) that uses the five DSDCs, and MemNet_C_. As shown in this table, the proposed method provided slightly higher or comparable denoising quality while it still has a much smaller computational complexity than the MemNet_C_, which will be analyzed in a later paragraph.

In the Pro_w/o_D, since only the *Loss_residual_* was used for the loss function, there was a problem in that some weak structural information could not be effectively preserved. Compared to the Pro_w/o_D, the Pro_wtih_D that uses *Loss_struct_* in addition to *Loss_residual_*, and the *D* provided slightly lower PSNR and SSIM values, but provided higher VIF and FSIM values that more accurately estimated the fidelity of structural information between the ground-truth and resultant images. To evaluate the utilization effectiveness of the *α*, we compared the denoising performances of the Pro_wtih_D that adjusts *α* value by using the results of the *D* as in Equation (8) and Pro_wtih_D without *α* that fixes the value of *α* to 1. As shown in Table 5, the Pro_wtih_D without *α* provided lower VIF and FSIM values that indicate the quality of structural information preservation than those of the Pro_wtih_D. This is because the *D*-based *α*-value adjustment allows training to be performed in the direction optimized for the characteristics of the input training data and the *G*.

**Figure 5:**
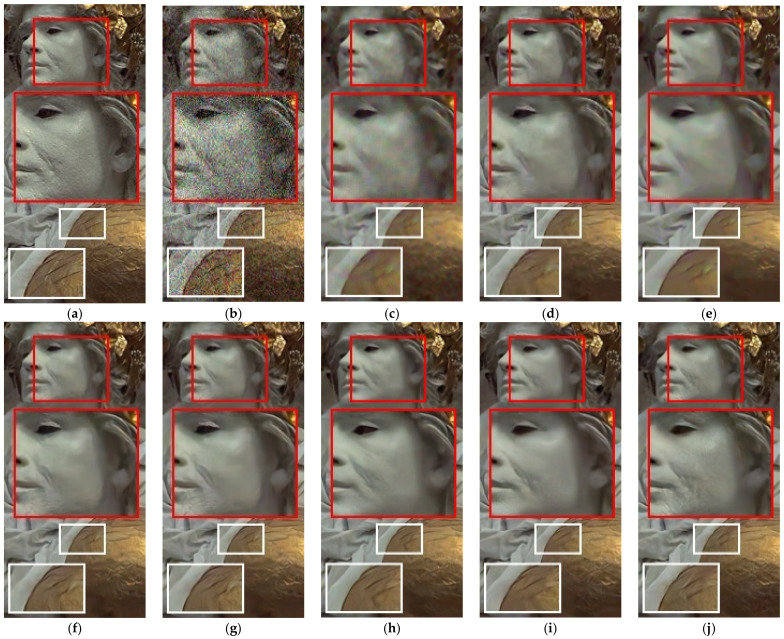
Denoised results of the benchmark and proposed methods for AWGN (noise level: σ_n_ = 35). (**a**) Original image (cropped from 4th (1st row), 15th (2nd row) and 22th (3rd row) images in Kodak image set) (**b**) Noisy image, (**c**) Image by NLM_C_, (**d**) Image by BM3D_C_, (**e**) Image by WNNM, (**f**) Image by DnCNN_C_, (**g**) Image by IRCNN_C_, (**h**) Image by MemNet_C_, (**i**) Image by the proposed method without *D* (Pro_w/o_D (DSDC^3^)), and (**j**) Image by the proposed method with *D* (Pro_wtih_D (DSDC^3^)).

**Figure 6:**
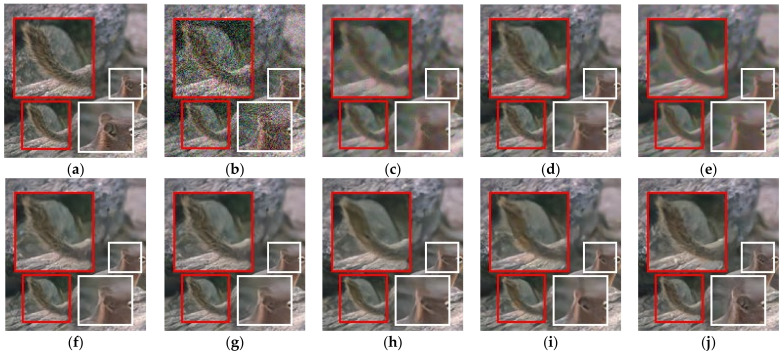
Denoised results of the benchmark and proposed methods for AWGN (noise level: σ_n_ = 35). (**a**) Original image with two enlarged patches (cropped from 20th image in CBSD68 image set) (**b**) Noisy image, (**c**) Image by NLM_C_, (**d**) Image by BM3D_C_, (**e**) Image by WNNM, (**f**) Image by DnCNN_C_, (**g**) Image by IRCNN_C_, (**h**) Image by MemNet_C_, (**i**) Image by the proposed method without *D* (Pro_w/o_D (DSDC^3^)), and (**j**) Image by the proposed method with *D* (Pro_wtih_D (DSDC^3^)).

Figure 5 and Figure 6 showed the resultant images by the benchmark methods, the Pro_w/o_D, and the Pro_wtih_D for noise level, *σ_n_* = 35. Figure 5 showed resultant images by the benchmark methods, the Pro_w/o_D, and the Pro_wtih_D. As in Figure 5, the deep learning-based methods provided the better qualities of noise elimination. Among the deep learning-based methods, the Pro_wtih_D most effectively preserved the small details that are spread throughout the statue face. In addition, the Pro_wtih_D showed an outstanding result in the preservation of rough textures around metal ball. In Figure 6, the Pro_wtih_D showed the best quality of detail preservation in animal’s tail and the ear compared with the benchmark methods. This is because the training of the Pro_wtih_D was performed in order to best reproduce the structural information of the restored image by the *G* as close as possible to the noise-free image by adjusting the strength of *Loss_struct_* depending on the result of the *D*.

### 3.2. Comparisons of Computational Complexity

As shown in Section 3.1, of the benchmark methods, DnCNN_C_, IRCNN_C_, and MemNet_C_ which are CNN-based denoisers, showed improved quality of denoising results than the other benchmark methods. In addition, the proposed method (Pro_w/o_D and Pro_wtih_D) is also a CNN denoiser. Thus, among the benchmark methods, we compared the computational complexities of the proposed method with DnCNN_C_, IRCNN_C_, and MemNet_C_. The Pro_w/o_D and the Pro_wtih_D have the same number of weights for their networks, since the use of the *D* is only applied during the training. This indicates that the computational complexity of the proposed *G* is equal to the computational complexity of the proposed method.

Since addition and subtraction operations require a very small amount of hardware resources compared to multiplication, the number of multiplications for convolution operations dominantly determines the computational complexity of the entire network. Hence, we compared the number of multiplications for each method for the comparison of the computational complexities of the benchmark and proposed methods, as shown in Table 6. As shown in this table, the proposed method (DSDC^3^) greatly reduced the number of multiplications to 20.96% and 62.12% compared to the DnCNN_C_ and IRCNN_C_, respectively, while providing higher PSNR and SSIM values for the various test image sets and noise levels. Compared with the MemNet_C_, the number of multiplications of the proposed methods using DSDC^3^ and DSDC^5^ are 2.34% and 3.96% of the MemNet_C_. In addition to the comparison of the number of multiplications, we compared the processing times (C_T_s) of the benchmark and the proposed methods. The C_T_s of each method were measured by using tensorflow on a PC with an Intel I7 7700 processor at 3.60 GHz, 16 GB DDR3s, and an Nvidia Titan X (Pascal) GPU. As shown in Table 7, although the proposed method (DSDC^3^) has fewer number of multiplications than the DnCNN_C_ and IRCNN_C_, the C_T_ of the proposed method was slightly larger than the DnCNN_C_ and IRCNN_C_. This is due to the fact that the proposed method has more convolution stages (because of DSC) in situations where each convolution layer was completely parallelized. However, the number of multiplications has the biggest effect on the cost for the HW implementation. Hence, the reduced number of multiplications of the proposed method could be an advantage in hardware design or CPU-based processing systems. In addition, the proposed method provided a noticeable improvement in denoising performance over the DnCNN_C_ and IRCNN_C_. Compared with the MemNet_C_, the proposed method showed a much lower C_T_ while providing the better or comparable denoising performance. This reduction of computational complexity of CNN can enhance the feasibility of CNN implementation in mobile applications and can increase energy efficiency.

## 4. Conclusions

In this paper, we proposed a novel GAN denoiser that uses heterogeneous losses, consisting of MSE-based loss and structural loss, for its training in order to improve the quality of detail preservation while maintaining the quality of noise suppression. In addition, a DSC-based module that utilizes the dilated convolution and symmetric skip connection was used for the proposed GAN denoiser in order to greatly reduce the computational complexity of the proposed network while maintaining or slightly increasing the denoising performance. In the proposed method, training was carried out so as to improve the quality of detail preservation using the GAN structure. By adjusting the strength of the proposed structural loss depending on the gradient fidelity between the original and restored images, which is calculated by the discriminator, we could reproduce the structural information most similar to that of the original image while maintaining the quality of noise suppression in smooth regions.

The advantages of the proposed method were verified on various test images and by noise levels. The proposed method showed the best denoising quality by providing various image quality indexes that were superior to those of the benchmark methods while greatly reducing the computational complexity.

## Figures and Tables

**Table 1:** The structure of a generator.

Layer	Operations	Dimension [S_R_, S_C_, F_D1_, F_D2_]
1st layer	P_conv + D_conv + bias + ReLU	P_conv: 1 × 1 × 24 × 96
		D_conv: 3 × 3 × 96 × 1
2nd~12th layers	P_conv + K dilated D_conv + BN + ReLU	P_conv: 1 × 1 × 96 × 96
		D_conv:3 × 3 × 96 × 1
13th layer	Conv + bias	Conv: 3 × 3 × 96 × 3
Total number of weights	116,640 (62% of the number of weights for IRCNN [1])	

**Table 2:** The structure of a discriminator.

Layer	Operations	Dimension [S_R_, S_C_, F_D1_, F_D2_]
1st layer	P_conv + D_conv + bias + ReLU	P_conv: 1 × 1 × 24 × 32
		D_conv: 3 × 3 × 32 × 1
2nd~12th layers	P_conv + K dilated D_conv + BN + ReLU	P_conv: 1 × 1 × 32 × 32
		D_conv:3 × 3 × 32 × 1
13th layer	Conv + bias	Conv: 3 × 3 × 32 × 3
14th layer	1024 dense (fully connected)	^1^ N × 1024
15th layer	1024 dense + sigmoid	1024 × 1

^1^ N: the number of pixels in the resultant image of the 13th layer.

**Table 3:** PSNRs, SSIMs, VIFs, and FSIMs of the benchmark and proposed methods.

Noise Level			*σ_n_* = 15							*σ_n_* = 25							*σ_n_* = 35						
Image Set (Number of Image Set)			Kodak (24)	CIPR_M (14)	CIPR_C (18)	IEC (20)	Football (90)	CBSD (68)	AVG	Kodak (24)	CIPR_M (14)	CIPR_C (18)	IEC (20)	Football (90)	CBSD (68)	AVG	Kodak (24)	CIPR_M (14)	CIPR_C (18)	IEC (20)	Football (90)	CBSD (68)	AVG
**Noisy** **images**		**PSNR [dB]**	24.610	24.607	24.607	24.609	24.611	24.609	24.609	20.172	20.177	20.170	20.172	20.174	20.172	20.173	17.249	17.253	17.251	17.250	17.249	17.249	17.250
		**SSIM**	0.682	0.654	0.627	0.606	0.663	0.726	0.660	0.485	0.455	0.418	0.391	0.449	0.544	0.457	0.360	0.334	0.298	0.270	0.319	0.421	0.334
		**VIF**	0.542	0.553	0.533	0.535	0.528	0.559	0.542	0.382	0.395	0.377	0.379	0.367	0.397	0.383	0.292	0.307	0.291	0.296	0.279	0.306	0.295
		**FSIM**	0.959	0.894	0.953	0.949	0.875	0.886	0.919	0.907	0.808	0.895	0.887	0.769	0.792	0.843	0.854	0.735	0.834	0.826	0.681	0.716	0.774
**Model-based optimization methods**	**NLM_C_**	**PSNR [dB]**	31.568	32.507	33.724	34.808	32.693	30.472	32.629	28.933	30.087	31.141	31.979	30.325	27.961	30.071	27.231	28.303	29.220	29.955	28.683	26.394	28.297
		**SSIM**	0.887	0.916	0.932	0.935	0.880	0.886	0.906	0.812	0.870	0.889	0.891	0.819	0.805	0.847	0.760	0.828	0.847	0.850	0.779	0.750	0.802
		**VIF**	0.472	0.529	0.514	0.518	0.435	0.490	0.493	0.327	0.404	0.388	0.386	0.310	0.349	0.361	0.255	0.332	0.318	0.317	0.253	0.276	0.292
		**FSIM**	0.972	0.963	0.981	0.977	0.921	0.922	0.956	0.945	0.939	0.963	0.957	0.883	0.867	0.926	0.925	0.921	0.946	0.938	0.869	0.839	0.906
	**BM3D_C_**	**PSNR [dB]**	34.415	33.997	35.793	37.745	35.444	33.513	35.151	31.824	31.945	33.697	35.298	32.976	30.705	32.741	30.044	30.436	32.074	33.489	31.282	28.880	31.034
		**SSIM**	0.934	0.940	0.958	0.964	0.943	0.937	0.946	0.893	0.914	0.938	0.942	0.904	0.890	0.913	0.853	0.889	0.917	0.920	0.866	0.845	0.882
		**VIF**	0.589	0.598	0.597	0.603	0.570	0.613	0.595	0.450	0.480	0.481	0.482	0.430	0.467	0.465	0.357	0.398	0.401	0.400	0.339	0.372	0.378
		**FSIM**	0.985	0.975	0.988	0.987	0.965	0.961	0.977	0.971	0.960	0.978	0.975	0.938	0.932	0.959	0.954	0.946	0.968	0.963	0.912	0.903	0.941
	**WNNM**	**PSNR [dB]**	32.484	33.657	34.932	36.286	33.847	31.272	33.746	30.117	31.446	32.647	33.858	31.454	28.772	31.383	28.653	29.997	31.026	32.172	29.953	27.276	29.846
		**SSIM**	0.909	0.933	0.949	0.952	0.914	0.907	0.927	0.858	0.905	0.924	0.925	0.862	0.849	0.887	0.817	0.880	0.900	0.900	0.824	0.800	0.854
		**VIF**	0.527	0.577	0.543	0.537	0.487	0.552	0.537	0.391	0.457	0.429	0.418	0.350	0.408	0.409	0.310	0.383	0.358	0.346	0.273	0.323	0.332
		**FSIM**	0.978	0.971	0.983	0.980	0.943	0.940	0.966	0.957	0.954	0.970	0.964	0.904	0.899	0.941	0.934	0.938	0.957	0.948	0.874	0.864	0.919
	**MC-WNNM**	**PSNR [dB]**	33.943	34.022	35.716	37.120	34.872	32.918	34.765	31.367	31.890	33.497	34.620	32.231	30.245	32.308	29.726	30.484	31.917	32.879	30.593	28.564	30.694
		**SSIM**	0.931	0.939	0.956	0.959	0.933	0.933	0.942	0.882	0.910	0.932	0.932	0.880	0.881	0.903	0.839	0.886	0.909	0.907	0.837	0.833	0.869
		**VIF**	0.579	0.597	0.586	0.577	0.538	0.604	0.580	0.434	0.477	0.467	0.452	0.386	0.455	0.445	0.345	0.402	0.392	0.374	0.300	0.363	0.363
		**FSIM**	0.983	0.974	0.987	0.984	0.958	0.959	0.974	0.965	0.957	0.975	0.970	0.921	0.924	0.952	0.946	0.942	0.964	0.955	0.890	0.892	0.931
**Discriminative** **learning methods** **(CNN-based methods**	**MLP**	**PSNR [dB]**	-	-	-	-	-	-	-	31.329	31.397	32.838	33.918	31.618	29.135	31.706	29.942	29.989	31.400	32.422	30.220	27.647	30.270
		**SSIM**	-	-	-	-	-	-	-	0.881	0.908	0.925	0.929	0.875	0.874	0.899	0.845	0.886	0.904	0.905	0.841	0.830	0.869
		**VIF**	-	-	-	-	-	-	-	0.378	0.446	0.392	0.368	0.366	0.415	0.394	0.304	0.373	0.323	0.299	0.288	0.331	0.320
		**FSIM**	-	-	-	-	-	-	-	0.909	0.937	0.935	0.930	0.904	0.913	0.921	0.881	0.920	0.919	0.912	0.877	0.882	0.899
	**Dn-** **CNN_C_**	**PSNR [dB]**	34.592	32.738	35.117	37.524	35.072	33.885	34.822	32.142	31.306	33.337	35.304	32.868	31.224	32.697	30.572	30.220	32.032	33.744	31.437	29.577	31.264
		**SSIM**	0.939	0.936	0.956	0.963	0.939	0.942	0.946	0.901	0.913	0.936	0.942	0.898	0.902	0.915	0.867	0.894	0.918	0.922	0.865	0.865	0.888
		**VIF**	0.598	0.587	0.582	0.597	0.560	0.627	0.592	0.461	0.478	0.470	0.478	0.423	0.485	0.466	0.375	0.406	0.399	0.402	0.342	0.397	0.387
		**FSIM**	0.985	0.972	0.987	0.985	0.960	0.964	0.976	0.971	0.957	0.977	0.973	0.932	0.938	0.958	0.957	0.944	0.967	0.962	0.908	0.915	0.942
	**IR-** **CNN_C_**	**PSNR [dB]**	34.686	34.146	35.785	37.659	35.309	33.855	35.240	32.154	32.096	33.716	35.346	32.964	31.140	32.903	30.552	30.690	32.275	33.750	31.476	29.475	31.370
		**SSIM**	0.939	0.940	0.958	0.964	0.942	0.942	0.947	0.902	0.917	0.938	0.943	0.902	0.900	0.917	0.868	0.896	0.920	0.923	0.867	0.863	0.889
		**VIF**	0.598	0.605	0.591	0.599	0.567	0.625	0.598	0.462	0.490	0.477	0.480	0.429	0.480	0.470	0.374	0.413	0.401	0.399	0.341	0.392	0.387
		**FSIM**	0.985	0.975	0.988	0.985	0.963	0.964	0.977	0.972	0.961	0.978	0.974	0.936	0.937	0.960	0.958	0.947	0.969	0.962	0.910	0.913	0.943
	**Mem-** **Net_C_**	**PSNR [dB]**	34.841	34.414	35.540	37.731	35.435	33.794	35.293	32.474	32.594	34.100	35.604	33.308	31.354	**33.239**	30.846	31.152	32.643	34.042	31.718	29.689	**31.682**
		**SSIM**	0.940	0.943	0.959	0.964	0.943	0.943	**0.949**	0.906	0.921	0.942	0.945	0.910	0.905	**0.922**	0.872	0.900	0.923	0.925	0.872	0.868	**0.893**
		**VIF**	0.606	0.615	0.598	0.605	0.577	0.630	0.605	0.478	0.506	0.492	0.495	0.448	0.495	**0.486**	0.389	0.428	0.416	0.413	0.353	0.403	**0.400**
		**FSIM**	0.986	0.977	0.988	0.986	0.965	0.965	0.978	0.974	0.964	0.980	0.978	0.944	0.942	**0.964**	0.960	0.951	0.971	0.965	0.916	0.918	**0.947**
	**Pro_w/o_D** **(DSDC^3^)**	**PSNR [dB]**	34.870	34.405	35.995	37.886	35.514	34.004	**35.446**	32.319	32.337	33.913	35.526	33.138	31.290	**33.087**	30.785	31.070	32.549	34.052	31.679	29.666	**31.634**
		**SSIM**	0.941	0.943	0.959	0.965	0.944	0.944	**0.949**	0.903	0.918	0.939	0.943	0.904	0.903	**0.918**	0.872	0.900	0.923	0.926	0.870	0.867	**0.893**
		**VIF**	0.607	0.615	0.601	0.607	0.581	0.632	**0.607**	0.467	0.491	0.480	0.481	0.432	0.488	0.473	0.382	0.421	0.409	0.405	0.344	0.400	0.394
		**FSIM**	0.986	0.977	0.988	0.986	0.966	0.966	**0.978**	0.972	0.961	0.979	0.975	0.938	0.940	0.961	0.959	0.950	0.970	0.963	0.911	0.916	0.945
	**Pro_wtih_D** **(DSDC^3^)**	**PSNR [dB]**	34.796	34.251	35.868	37.743	35.386	33.958	**35.334**	32.275	32.296	33.832	35.409	33.086	31.250	33.025	30.729	30.957	32.470	33.940	31.654	29.631	31.564
		**SSIM**	0.940	0.942	0.958	0.964	0.942	0.943	**0.948**	0.902	0.917	0.938	0.941	0.902	0.902	0.917	0.870	0.898	0.921	0.924	0.870	0.867	**0.892**
		**VIF**	0.608	0.615	0.603	0.607	0.580	0.632	**0.607**	0.469	0.495	0.485	0.486	0.437	0.489	**0.477**	0.383	0.421	0.410	0.409	0.350	0.400	**0.396**
		**FSIM**	0.986	0.977	0.988	0.986	0.965	0.966	**0.978**	0.973	0.963	0.979	0.975	0.940	0.941	**0.962**	0.959	0.951	0.969	0.964	0.916	0.919	**0.946**

DSDC^3^: consists of three DSDCs as shown in Figure 1a.

**Table 4:** PSNR and SSIM values of the MemNet_C_ and the proposed method using the increased number of DSDCs (DSDC^5^ network).

Image Set			Kodak	CIPR_M	CIPR_C	AVG
*σ_n_* = 25	**MemNet_C_**	**PSNR [dB]**	32.474	32.594	34.100	33.06
		**SSIM**	0.906	0.921	0.942	0.92
	**Pro_w/o_D_** **DSDC^5^**	**PSNR [dB]**	32.424	32.502	34.052	32.99
		**SSIM**	0.905	0.920	0.942	0.92
*σ_n_* = 35	**MemNet_C_**	**PSNR [dB]**	30.846	31.152	32.643	31.55
		**SSIM**	0.872	0.900	0.923	0.90
	**Pro_w/o_D_** **DSDC^5^**	**PSNR [dB]**	30.860	31.168	32.654	31.56
		**SSIM**	0.874	0.901	0.925	0.90

DSDC^5^: consists of five DSDCs.

**Table 5:** PSNRs, SSIMs, VIFs, and FSIMs of the Pro_wtih_D and Pro_wtih_D without α in Equation (8).

Image Set			Kodak	CIPR_M	CIPR_C	AVG
*σ_n_* = 15	**Pro_wtih_D**	**PSNR [dB]**	34.796	34.251	35.868	34.972
		**SSIM**	0.940	0.942	0.958	0.947
		**VIF**	0.608	0.615	0.603	** **0.609** **
		**FSIM**	0.986	0.977	0.988	** **0.984** **
	**Pro_wtih_D** **without α**	**PSNR [dB]**	34.828	34.398	35.956	35.061
		**SSIM**	0.940	0.942	0.959	0.947
		**VIF**	0.603	0.611	0.595	0.603
		**FSIM**	0.986	0.976	0.988	0.983
*σ_n_* = 25	**Pro_wtih_D**	**PSNR [dB]**	32.275	32.296	33.832	32.801
		**SSIM**	0.902	0.917	0.938	0.919
		**VIF**	0.469	0.495	0.485	** **0.483** **
		**FSIM**	0.973	0.963	0.979	** **0.972** **
	**Pro_wtih_D** **without α**	**PSNR [dB]**	32.286	32.351	33.871	32.836
		**SSIM**	0.903	0.918	0.939	0.920
		**VIF**	0.466	0.493	0.480	0.480
		**FSIM**	0.972	0.962	0.979	0.971
*σ_n_* = 35	**Pro_wtih_D**	**PSNR [dB]**	30.726	30.957	32.473	31.385
		**SSIM**	0.871	0.898	0.921	0.897
		**VIF**	0.383	0.421	0.411	** **0.405** **
		**FSIM**	0.959	0.951	0.969	** **0.960** **
	**Pro_wtih_D** **without α**	**PSNR [dB]**	30.747	30.968	32.468	31.394
		**SSIM**	0.871	0.899	0.922	0.897
		**VIF**	0.381	0.420	0.408	0.403
		**FSIM**	0.958	0.950	0.969	0.959

**Table 6:** Comparison of the number of multiplications.

Parameter	DnCNN_C_	IRCNN_C_		MemNet_C_
The number of weights	(3 × 3 × 3 × 64) + (3 × 3 × 64 × 64 × 15) + (3 × 3 × 64 × 3) = 556416	(3 × 3 × 3 × 64) + (3 × 3 × 64 × 64 × 5) + (3 × 3 × 64 × 3) = 187776		3 × 3 × 3 × 64 + 3 × 3 × 64 × 64 × (2 × 6 × 6 + 6) + 3 × 3 × 64 × 64 × (7 + 8 + 9 + 10 + 11 + 12) = 4978368
**Parameter**	**Pro_w/o_D (DSDC^3^)**		**Pro_w/o_D (DSDC^5^)**	
The number of weights	1 × 1 × 24 × 96 + 12 × (3 × 3 × 96) + 11 × (1 × 1 × 96 × 96) + 3 × 3 × 96 × 3 = 116640		1 × 1 × 24 × 96 + 20 × (3 × 3 × 96) + 19 × (1 × 1 × 96 × 96) + 3 × 3 × 96 × 3 = 197280	

**Table 7:** Comparison of the processing time per pixel (C_T_).

Method	DnCNN_C_	IRCNN_C_	MemNet_C_	Pro_w/o_D (DSDC^3^)	Pro_w/o_D (DSDC^5^)
C_T_ (MS)	0.16	0.08	1.29	0.21	0.32

## References

[1] Zhang K., Zuo W., Gu S., Zhang L. Learning deep CNN denoiser prior for image restoration. Proceedings of the IEEE Conference on Computer Vision and Pattern Recognition (CVPR).

[2] Perona P., Malik J. (1990). Scale-space and edge detection using anisotropic diffusion. IEEE Trans. Pattern Anal. Mach. Intell..

[3] Rudin L.I., Osher S., Fatemi E. (1992). Nonlinear total variation based noise removal algorithms. Phys. D Nonlinear Phenom..

[4] Tomasi C., Manduchi R. Bilateral filtering for gray and color images. Proceedings of the IEEE International Conference on Computer Vision (ICCV).

[5] Buades A., Coll B., Morel J.M. A non-local algorithm for image denoising. Proceedings of the IEEE Conference on Computer Vision and Pattern Recognition (CVPR).

[6] Dabov K., Foi A., Katkovnik V., Egiazarian K. (2007). Image denoising by sparse 3-D transform-domain collaborative filtering. IEEE Trans. Image Process..

[7] Gu S., Zhang L., Zuo W., Feng X. Weighted nuclear norm minimization with application to image denoising. Proceedings of the IEEE Conference on Computer Vision and Pattern Recognition (CVPR).

[8] Xu J., Zhang L., Zhang D., Feng X. Multi-channel Weighted Nuclear Norm Minimization for Real Color Image Denoising. Proceedings of the 2017 IEEE International Conference on Computer Vision (ICCV).

[9] Burger H.C., Schuler C.J., Harmeling S. Image denoising: Can plain neural networks compete with BM3D?. Proceedings of the IEEE Conference on Computer Vision and Pattern Recognition (CVPR).

[10] Zhang K., Zuo W., Chen Y., Meng D., Zhang L. (2017). Beyond a Gaussian denoiser: Residual learning of deep CNN for image denoising. IEEE Trans. Image Process..

[11] Tai Y., Yang J., Liu X., Xu C. MemNet: A Persistent Memory Network for Image Restoration. Proceedings of the IEEE International Conference on Computer Vision (ICCV).

[12] Zhang K., Zuo W., Zhang L. (2018). Ffdnet: Toward a fast and flexible solution for cnn-based image denoising. IEEE Trans. Image Process..

[13] Guo S., Yan Z., Zhang K., Zuo W., Zhang L. Toward convolutional blind denoising of real photographs. Proceedings of the IEEE Conference on Computer Vision and Pattern Recognition (CVPR).

[14] Chen J., Chen J., Chao H., Yang M. Image blind denoising with generative adversarial network based noise modeling. Proceedings of the IEEE Conference on Computer Vision and Pattern Recognition (CVPR).

[15] Yin J., Chen B., Li Y. (2018). Highly Accurate Image Reconstruction for Multimodal Noise Suppression Using Semisupervised Learning on Big Data. IEEE Trans. Multimed..

[16] Hou X., Luo H., Liu J., Xu B., Sun K., Gong Y., Liu B., Qiu G. Learning Deep Image Priors for Blind Image Denoising. Proceedings of the IEEE Conference on Computer Vision and Pattern Recognition Workshops (CVPRW).

[17] Goodfellow I., Pouget-Abadie J., Mehdi M., Xu B., Warde-Farley D., Ozair S., Courville A., Bengio Y. Generative adversarial nets. Proceedings of the Advances in Neural Information Processing Systems.

[18] Sifre L. (2014). Rigid-Motion Scattering for Image Classification. Ph.D. Thesis.

[19] Cho S.I., Kang S.-J. (2019). Gradient Prior-aided CNN Denoiser with Separable Convolution-based Optimization of Feature Dimension. IEEE Trans. Multimed..

[20] Ioffe S., Szegedy C. Batch normalization: Accelerating deep network training by reducing internal covariate shift. Proceedings of the International Conference on Machine Learning (ICML).

[21] Krizhevsky A., Sutskever I., Hinton G.E. (2012). Imagenet classification with deep convolutional neural networks. Advances in Neural Information Processing Systems.

[22] Yu F., Koltun V. Multi-scale context aggregation by dilated convolutions. Proceedings of the International Conference on Learning Representations (ICLR).

[23] He K., Zhang X., Ren S., Sun J. Deep residual learning for image recognition. Proceedings of the IEEE Conference on Computer Vision and Pattern Recognition (CVPR).

[24] CIPR Database. http://www.cipr.rpi.edu/resource/stills/index.html.

[25] Jones K. (2008). Methods of measurement for the power consumption of audio, video and related equipment. ENERGY STAR Program. Requirements for Displays.

[26] Football Sequences. http://media.xiph.org/video/derf/.

[27] Cho S.I., Kang S.-J. (2018). Geodesic path-based diffusion acceleration for image denoising. IEEE Trans. Multimed..

[28] Kingma D., Ba J. A method for stochastic optimization. Proceedings of the International Conference on Learning Representations (ICLR).

[29] Fowlkes C.C., Martin D.R., Malik J. (2007). Local figure–ground cues are valid for natural images. J. Vis..

[30] Deng J., Dong W., Socher R., Li L.-J., Li K., Fei-Fei L. Imagenet: A large-scale hierarchical image database. Proceedings of the IEEE Conference on Computer Vision and Pattern Recognition (CVPR).

[31] Ma K., Duanmu Z., Wu Q., Wang Z., Yong H., Li H., Zhang L. (2017). Waterloo exploration database: New challenges for image quality assessment models. IEEE Trans. Image Process..

[32] Abadi M., Agarwal A., Barham P., Brevdo E., Chen Z., Citro C., Corrado G.S., Davis A., Dean J., Devin M. (2016). TensorFlow: Large-Scale Machine Learning on Heterogeneous Systems. arXiv.

[33] Wang Z., Bovik A.C., Sheikh H.R., Simoncelli E.P. (2004). Image quality assessment: From error visibility to structural similarity. IEEE Trans. Image Process..

[34] Sheikh H.R., Bovik A.C. (2006). Image information and visual quality. IEEE Trans. Image Process..

[35] Zhang L., Zhang L., Mou X., Zhang D. (2011). FSIM: A feature similarity index for image quality assessment. IEEE Trans. Image Process..

[36] Li T.H., Li Z., Han T.Y., Rahardja S., Yeo C. (2013). A perceptually relevant MSE-based image quality. IEEE Trans. Image Process..

[37] Shao L., Yan R., Li X., Liu Y. (2014). From heuristic optimization to dictionary learning: A review and comprehensive comparison of image denoising algorithms. IEEE Trans. Cybern..

[38] Kang L.-W., Lin C.-W., Fu Y.-H. (2012). Automatic single-image-based rain streaks removal via image decomposition. IEEE Trans. Image Process..

[39] Huang T.-H. (2013). Enhancement of Backlight-Scaled Images. IEEE Trans. Image Process..

[40] Li S., Kang X. (2012). Fast multi-exposure image fusion with median filter and recursive filter. IEEE Trans. Consum. Electron..

[41] Zhang Y., Zhang Y., Zhang J., Dai Q. (2016). CCR: Clustering and collaborative representation for fast single image super-resolution. IEEE Trans. Multimed..

[42] Jiang J., Ma X., Chen C., Lu T., Wang Z., Ma J. (2017). Single image super-resolution via locally regularized anchored neighborhood regression and nonlocal means. IEEE Trans. Multimed..

[43] Du B., Zhang M., Zhang L., Hu R., Tao D. (2017). PLTD: Patch-based low-rank tensor decomposition for hyperspectral images. IEEE Trans. Multimed..

[44] Liu X., Zhao D., Xiong R., Ma S., Gao W., Sun H. (2011). Image interpolation via regularized local linear regression. IEEE Trans. Image Process..

